# Pathways linking car transport for young adults and the public health in Northern Ireland: a qualitative study to inform the evaluation of graduated driver licensing

**DOI:** 10.1186/s12889-017-4470-x

**Published:** 2017-06-07

**Authors:** Nicola Christie, Rebecca Steinbach, Judith Green, M. Patricia Mullan, Lindsay Prior

**Affiliations:** 1 Centre for Transport Studies, UCL, Gower Street, London, WC1E 6BT UK; 2 Faculty of Public Health and Policy, London School of Hygiene and Tropical Medicine, WC1H 9SH, London, UK; 3 Centre of Excellence for Public Health, Queen’s University, Belfast, BT7 1NN UK; 4 Present address: Division of Health & Social Care Research, Faculty of Life Sciences and Medicine, King’s College London, Addison House, London, SE1 1UL UK

**Keywords:** Graduated driver licensing, Driving, Focus groups, Logic model, Northern Ireland, Qualitative, Telematics, Transport

## Abstract

**Background:**

Novice drivers are at relatively high risk of road traffic injury. There is good evidence that Graduated Driving Licensing (GDL) schemes reduce collisions rates, by reducing exposure to risk and by extending learning periods. Legislation for a proposed scheme in Northern Ireland was passed in 2016, providing an opportunity for future evaluation of the full public health impacts of a scheme in a European context within a natural experiment. This qualitative study was designed to inform the logic model for such an evaluation, and provide baseline qualitative data on the role of private cars in health and wellbeing.

**Methods:**

Nine group interviews with young people aged 16–23 (*N* = 43) and two group interviews with parents of young people (*N* = 8) were conducted in a range of settings in Northern Ireland in 2015. Data were analysed using thematic content analysis.

**Results:**

Informal car-pooling within and beyond households led to routine expectations of lift provision and uptake. Experiences of risky driving situations were widespread. In rural areas, extensive use of farm vehicles for transport needs meant many learner drivers had both early driving experience and expectations that legislation may have to be locally adapted to meet social needs. Cars were used as a site for socialising, as well as essential means of transport. Alternative modes (public transport, walking and cycling) were held in low esteem, even where available. Recall of other transport-related public health messages and parents’ existing use of GDL-type restrictions suggested GDL schemes were acceptable in principle. There was growing awareness and use of in-car technologies (telematics) used by insurance companies to reward good driving.

**Conclusions:**

Key issues to consider in evaluating the broader public health impact of GDL will include: changes in injury rates for licensed car occupants and other populations and modes; changes in exposure to risk in the licensed and general population; and impact on transport exclusion. We suggest an important pathway will be change in social norms around offering and accepting lifts and to risk-taking. The growing adoption of in-car telematics will have implications for future GDL programmes and for evaluation.

## Background

Road traffic injury is a major and growing contributor to mortality and disability for young adults worldwide [1, 2]. Novice drivers are at high risk due to both inexperience and youth [3], which are independently associated with risk [4]. Risks of collision are particularly high for males [5], those carrying passengers [6–8], and those driving at night [5, 8]. Despite the United Kingdom (UK) having a relatively good record on road safety in general [9] drivers aged 17–24 years are still overrepresented in the ‘killed and seriously injured’ category compared with the numbers licenced to drive [10], and road injury is the leading cause of mortality for those aged 15–19 [11]. This high risk is also disproportionately shared, with young drivers from the most deprived areas significantly overrepresented in fatal crashes compared to those from more affluent areas [12].

### Graduated driver licensing

Graduated Driver Licensing (GDL) schemes have been an effective intervention for reducing death and serious injury to young drivers in many settings [13–17]. The first, full GDL scheme was implemented in New Zealand in 1987, and they have now been widely adopted across Australasia [13, 14] and north America, including most states in the USA [15, 16]. Schemes vary widely, but they typically include phased licences (which initially permit driving only while supervised by a fully licensed person, then only with certain restrictions), which must be held for a minimum period of time [17]. There are two key mechanisms through which they operate: improving skills through mandated periods of supervised driving; and reducing exposure to known high risk conditions, such as carrying teenage passengers and driving at night. There is now a substantial body of evidence [18] identifying the positive impact of such schemes on collision rates for young drivers [19–23].

However, there is considerable variation across schemes internationally and over time in the minimum age for licences, the length of time a provisional (intermediate, or restricted) licence must be held before driving unsupervised, the restrictions imposed on novice drivers and the enforcement of infractions [18, 22–24]. Given the heterogeneity of schemes, a Cochrane review of 2011 [22] could not make an overall estimate of effect of GDL, but concluded that reductions in collision rates were seen in all jurisdictions and for all crash types, with reductions in collisions involving injury for teenage drivers by a median of 20% beyond the first year post GDL. Effects are more robust for 16 and 17 year old drivers than for those over 18 [24]: a limitation is that schemes only in force for young novice drivers may delay licensing [25]. There is some evidence of a dose-response type relationship, in that severity of restrictions (such as earlier curfews) and longer periods of training are identified with lower risks [21, 26]. In general, identifying which components of GDL are effective has been challenging [16, 21].

Existing evidence has largely has largely come from jurisdictions outside Europe, and has addressed only crash or injury rates [22, 24, 27, 28]. However, the public health effects of GDL schemes may be wider than those that relate to car injuries. Collisions are not the only outcome affected by restrictions on novice drivers: one review [23] highlighted commonly cited concerns raised by policy makers and other stakeholders about the potential negative consequences for young drivers from restrictions on mobility, such as access to employment, especially in rural areas and for less affluent young adults, for whom transport exclusion is already a deterrent to work opportunities in UK settings [29]. In settings outside North America and Australasia, where transport systems differ, and car ownership rates may be lower,^1^ there may also be different implications of passenger restrictions from mode switching, or increases in numbers of cars on the roads, if (for instance) it becomes less possible to have one ‘designated driver’ for groups of young adults socialising together.

### Study context: Northern Ireland

Northern Ireland has a population of 1.8 million, with around 35% living in rural areas. In 2015, around 42% of 17–20 year olds held a full private car driving licence.^2^ Car ownership rates are around 640 per 1000 population aged over 17 (including farm vehicles) [30].^3^ A Road Traffic (Amendment) Bill was passed by the Northern Ireland Assembly in 2016, which introduced (among other measures) a Graduated Driver Licensing Scheme, the first in the UK. In summary, this will introduce a mandatory six month period of training, to be evidenced by a log book, before the full licence can be applied for, and (for those under 24 years old) restrictions to no more than one young (aged 14–20) passenger between the hours of 11 pm and 6 am for 6 months post-test. Implementation is planned for 2018, following further consultation and secondary legislation. This will replace the system in place when the fieldwork for this study was conducted, in which a provisional driving licence for a motor car (a vehicle of up to eight seats) can be held from age 17 (or from 16 for some young people with disabilities). This entitles the learner to drive supervised (by someone aged at least 21, who has held a full driving licence for at least three years) in a car displaying visible ‘L’ plates. Entitlement to drive unsupervised on a full licence is conditional on first passing a driving theory test, and then passing a practical driving test, which includes questions on safety and assessment of driving skills. There is no mandatory minimum period of time for holding a provisional licence, but visible ‘R’ plates must be displayed and speed limit restrictions observed for one year. Northern Ireland shared, at the time of the study, an open land border with the Republic of Ireland, which had similar licensing arrangements, except that novice drivers who have passed a test must display ‘N’ plates for two years post-test.

Graduated Driving Licensing schemes are not common in Europe [31, 32], and no other schemes are proposed (currently) in other countries in the UK, despite policy calls for their introduction to reduce the burden of injury [33]. These calls highlight that major risks for novice drivers are similar in the UK to countries where GDL has been effective. In Great Britain, for instance, around 25% of crashes to young drivers occur between 9 pm and 6 am, and 24% occur with at least one 15–24 passenger present [8, 11, 33]. A DfT- commissioned review to examine the evidence published since the 2011 Cochrane review concluded that there was compelling evidence that GDL could reduce young driver crash rates in the UK [23]. However, it also highlighted some commonly cited barriers to the implementation of GDL, namely issues around enforcement and compliance and effects on mobility and employment, especially in remote areas [23]. These issues had been raised as rationales for not considering GDL schemes in other UK jurisdictions [32].

The proposed scheme in Northern Ireland presents, then, a useful opportunity to design an evaluation of GDL in a European context, where there have been few evaluations to date, and to design an evaluation of the wider public health impacts of such a scheme. Implementation of GDL in Northern Ireland would be a natural experiment, allowing comparisons of key outcomes (such as injury rates) over time across the exposed and non-exposed countries of the UK.

### Rationale for study

To enable a robust and useful future evaluation, this present study aimed to provide: 1) a qualitative data set that could be utilised for comparative purposes in future evaluations (to compare, for instance, changes in social norms around risky driving); and 2) to explore the current role of private cars in the lives of young people such that a logic model of potential pathways to health impact could be developed. We therefore undertook a qualitative study of young people’s driving practices (e.g. being a driver or not, being a passenger, learning to drive) to understand and document current (‘before’ intervention) practice, and to identify pathways potentially affected by the proposed intervention. These were then mapped as a logic model, in the light of broader literature and consultation with stakeholders such as policy makers, which outlined key potential causal pathways that link the intervention (GDL) to determinants of public health.

One challenge in any evaluation of such natural experiments is that transport is a complex system, in which change is likely to result not only from the intervention, but also from predictable and less predictable confounders which are difficult to control for in most research designs [34]. Later licensing in young adults across many highly motorised countries [35], for instance, is likely to change the age structures of populations of licensed drivers over time, and also potentially change the implications of car access and ownership for young adults. There have also been rapid developments of technologies used by car insurers to introduce dynamic premiums. In-car ‘telematics’ [36] are technologies that use Global Positioning System technology and sensors to monitor driving distance, speed, and style (e.g. sudden braking or poor cornering). These devices have been found to influence driving behaviour [37] but there is to date (to our knowledge) no peer reviewed evidence on their effects on outcomes such as road injury. Given the prohibitively high costs of car insurance for young adults in the UK, in-car telematics have become popular. Clearly, if widely adopted, telematics significantly change the landscape for public health interventions in road injury reduction. Telematic technologies potentially delegate some enforcement of GDL type restrictions; as such they may be more effective than legislative change. They also potentially impact on driving behaviour and risks in both GDL exposed and non-exposed groups (such as novice drivers in Northern Ireland and those elsewhere in the UK). In-car technologies have not been used to date as part of GDL schemes or for evaluation. However, by the time the proposed scheme in Northern Ireland has been implemented, they may well have become widely adopted. We were therefore also interested in the role of telematics in current decisions.

## Methods

### Sample

To inform a logic model, this study aimed to map key potential pathways by which private car transport is related to public health and to identify those pathways potentially affected by the introduction of GDL schemes. The focus was on the population targeted by GDL; those aged under 24 years, and their parents. The youngest (those under 17 years) were at an age where they were anticipating taking driving lessons and accessing cars; those over 17 were legally able to drive but were still novices, with no more than a few years’ (formal) experience. All were at an age that would be affected as potential passengers by proposed legislation (under 24 years). Nine focus groups with ‘natural groups’ of young people aged 16–23 (*N* = 43 participants; group size ranged from three to eight) and two with parents of young adults (*N* = 8 participants) were used to access collaborative ‘stories’ and tacit knowledge around car travel. Natural groups, who know and interact with each other outside the research setting, have many advantages over individual interviews for understanding practices in context, including: providing access to group norms as participants interact within the group [38]; enabling participants to raise their own questions and analyses concerning ‘the way things are’; and encouraging more open discussion about potentially sensitive issues, such as risk taking while driving.

Sampling was purposive rather than statistical, with an aim of including groups from diverse settings and with a wide range of circumstances that might influence transport experiences, and to ‘over-sample’ from those in more deprived areas, where young drivers are at highest risk. First, geographic areas were sampled to include a range of urban, rural and more and less deprived settings (Table 1). Within those areas, we invited specific groups to include maximum variation in terms of gender, religious community, education level, and local transport availability (see Table 2). Analytical saturation was achieved after 11 transcripts were analysed.

**Table 1:** Sampling grid for group interview settings

	Deprivation level		
	*Low*	*Mid*	*High*
*Rural/Suburban*	D	A,B	G, J
*Urban*	E, K	I	C, F, H

Note: A-I denote a group of young people; J and K denote a group of parents

**Table 2:** Demographic characteristics of young adult participants

	N (participants)
Age	
16–17	18
18–19	8
20–21	13
22–23	4
Religious community	
Protestant	11
Catholic	20
Other Christian	2
No religion	8
Not stated	2
Ethnicity	
White British	16
White Irish	25
White other	2
Gender	
Male	23
Female	20
NIMDM ^a^ of postcode (tercile)	
Low	12
Mid	10
High	20
Not stated	1
TOTAL	43

Note: ^a^Northern Ireland Multiple Deprivation Measure

### Ethics

Northern Ireland is a relatively small country, and we included some areas with very few residents. We have therefore provided summary details only of the group settings, and not included demographic details of the parents. To preserve confidentiality, all personal and place names (except Belfast) are pseudonyms, and other identifying details have been removed. Extracts in this paper are tagged with a letter to indicate the group (A - I are young people; J and K are parents) and speaker (I, interviewer; M or F, male or female participant respectively). Individuals gave written consent for participation.

### Data collection and analysis

Topic guides aimed to elucidate stories on: travel to education, work, training and social activities; experiences of driving and being a car passenger; decisions around learning and licensing; and views of enforcement and telematics. Fieldwork took place between June and October 2015, with all groups facilitated by one of the authors (MPM or RS; one from Northern Ireland, one not), in either community facilities (such as local halls) or private houses. All groups were recorded, with permission, and transcribed in full. Data were analysed using a deductive thematic content analysis focused on the role of cars and driving on the determinants of health, exposure to road injury risk, and the likely effects of GDL restrictions and telematics-based insurance products. Coding was undertaken by two authors (RS and JG). Topic guides and coding schemes are available from the LSHTM Data Compass repository (10.17037/DATA.33).

## Results

### The importance of private cars and informal car-pooling

The relative importance of private car use for young adults’ access to education, training, work and social life depended largely on local transport infrastructure. In areas of thinly dispersed settlements, where workplaces and colleges are scattered, and public transport links poor or non-existent, access to private cars was considered indispensable for securing essential determinants of health: work and apprenticeships; goods; health services and amenities such as sports clubs:

F: Yeah. I find that because I don't drive, around here in the summers there's not really an option to work, even if it is only in the town. (A)

Young adults who could not drive or did not have access to a car were therefore heavily reliant on a local and informal system of lift sharing. Parents were typically primarily responsible for providing transport, although the networks of those sharing lifts also extended beyond the household to workmates and neighbours. Group J, for example, described the range of transport needs of their older children, which they met largely by providing lifts themselves:

M1: Sports training or matches and to get buses to matches or to get a bus to go out…

F1: To see their friends -

M1:- and to pick them up from a bus again later on at night or drop them off at a friend’s house and pick them up again later.

M2: In and out of the town. I took Danny to Drumcreagh over the weekend and Ellen’s in and out of the town (J)

The logistics of providing multiple transport needs within and beyond households often entailed complex rotas for car sharing, or managing other commitments around transport availability:

M: My sister is the main one with priority because she's in uni at the minute. If she's on placement and she will have that car at her disposal. Then it's mum and then me. I'm the last priority. That's why I have to organise it week by week. It's like an actual full on timetable who gets it. (G)

F: Yeah, my sister works in the same place, so usually we get the same shifts in work and she'd take me, and if not, my mum would take me or I'd get a taxi. (D)

Lifts were discussed in ways suggesting that contributing to this communal provision was a routine social commitment: anyone with a licence and a car would be expected to help out. However, if lifts to school or organised leisure activities (such as sports clubs) were expected from parents, the provision of non-essential transport, such as to friends’ houses, was perceived as an added burden, with young adults describing themselves as reluctant to make too many demands:

F: Ah [laughter] you feel lousy if you need to get picked up from somewhere at like 1 am and your parents have to like stay up or whatever, you don't know how to get home or, you know, that sort of thing. Just having to depend on them the whole time for lifts is not fair. [Laughter] (A)

### Alternatives to private car use

If access to private car transport was indispensable in rural areas, it was also often viewed as essential in urban settings. Even where reasonably reliable and accessible bus services had “most stops are within walking distance” (E), buses were often presented as a transport mode of last resort:

M: Usually either my sister or my father, just whoever is handy to give me a lift in, and then sometimes I take the bus into town (E)

Apart from limitations in availability, accessibility, frequency and comfort of facilities, public transport in urban areas might also entail perceived potential risks, or threats, of violence if travelling through an area that was identified as belonging to a different community. Participants referred, in particular, to the period around the 12th July,^4^ when inter-community tensions are often high, as a time when cars would feel safer:

M: You could be in the bus in one area and you're in a complete and utterly different area that you might not want to be in.

F: Driving, you can go on your own route, on a bus you have to stick to their route

M: And then there might be someone that sees you and knows that you're not [from their area]

F: Sees you in their area and then it's just trouble.

M: Yeah, it happens all the time, especially around this time of the year as well.

I: Right, around the 12th. (F)

The exceptions to largely negative views of public transport were a few students in an urban area with a good bus and local train system (H) who received a discount card: one noted that even if she were able to drive, the bus would be easier for most routine journeys, as there were no issues of parking. However, even this group discussed how you would “obviously get in the car” (H) for going beyond the city, or for getting home from social activities late at night when the buses did not run.

If buses provided an available, if unappealing, alternative to private cars in urban areas, shorter distances also made walking a feasible way to access work, education and social life. However, walking, like taking the bus, was also described largely as a mode utilised only “Because you have to” (C) when no car was available. Disincentives included changeable weather conditions and (particularly in rural areas) unsafe roads with fast moving traffic. Cycling was reported as even less appealing. A range of pragmatic reasons were offered for the low uptake of cycling as a way of getting around, including inhospitable weather and roads. More fundamental barriers lay in the normative framing of cycling as inherently inappropriate (indeed laughable) as an adult mode of transport, even if familiar as a potential option that might be used in other places, such as “the UK” (referring to other UK countries), or as a sporting activity:

F: You wouldn't bike anywhere. It’s a bit of a secret hobby. No … middle-aged man wants to be seen in their frilly Lycra and stuff. [Laughter] I think that's a big part of it. It's so different in the UK where it's like, "oh, I just cycled into work", whereas around here it's like… [Laughter] (A)

As one participant summarised: “I wouldn’t be seen dead on a bike… you’d never live it down!” (F).

### The incentives to learn to drive: Contributions and independence

In a context where private car use is considered essential, and where families often have to manage complex arrangements of shared lifts, young adults were often directly encouraged to learn to drive in order to contribute to transport provision:

M: We got them started very young just and when it came to driving age then they were straight in to lessons. There was somebody else who could scoot into the town to do messages [i.e. shopping]; it was brilliant. That’s why we wanted the [children] driving. (J)

Parents were the most enthusiastic about early licensing: it was largely they who paid for formal lessons, and who often both paid for and managed insurance and other costs, particularly for offspring still in education. For young people, coming up against the constraints of existing informal car-sharing arrangements could provide the final incentive to learn or to get a car:

F: Well, it actually took me a while to get mine, because my sister was there and I never really like needed [a car]. Like, anywhere she was going, I was going too. And then she moved out so then I decided it was about time I got my licence, and now that I have it, like, I feel that you can just go whenever you want. (D)

Although parents’ encouragement and family circumstances might provide the immediate incentive or rationale, this appeared to be largely in line with young adults’ own expectations, which were to both learn to drive, and have access to car, as soon as possible.

I: Why is that? Why did you go for it so young?

M: Well, I needed it for work, to get about, bits and places. I've actually been driving a tractor since I was 16 on a provisional and then when I was 17 I got my licence within five weeks to get fro and back to work. (D)

Beyond the pragmatic advantages of being able to contribute to, rather than being a burden on, informal car-pooling arrangements, driving was also a more symbolic marker of adulthood. Holding a driving licence, and if possible owning a car, had a status beyond that of instrumental value in that it signalled ‘independence’ in both practical and social senses. This was a view expressed by both parents and young adults across all groups:

F: I mean, our thing was you should learn to drive and then you've got it and that's, you know, if you do end up that you're in a job and you need it then it's already taken care of. (K)

F: I'm always relying on my mum and dad to transport me, and, you know, I don't really like relying on them. I would rather be independent and get my own way and not rely on anyone. (D)

### Spins and stories: The car as a site for socialising

In addition to signalling adult status, there were a range of other social benefits that accrued directly from being able to drive, or knowing peers who could. For groups of friends, access to a car provided not only the ability to get from A to B, but also a place for socialising in itself, particularly in rural areas where there were few alternatives. The importance of sociability meant most preferred giving lifts to friends over driving alone:

M: It's just chatting to your friends is good.

F: The car is a really good place to talk to people, because you don't have to look them in the eye, you don't have to worry about eye contact or what you're thinking. You're both facing forward, you can really get into the heart of matters, I think. I enjoy it, in any case. (A)

M1: I don’t mind. I like a bit of company in the car. You can’t be driving on your own. You’d be bored […]

M2: Yeah, it’s better craic [fun] having passengers. (B)

Cars were, not, then, merely a means of transport to places of social activity: they also provided a valued social space in themselves. Indeed, the ‘spin’ (a drive around, with no particular destination in mind) was a much cited source of entertainment. Typically, stories of such ‘spins’ or ‘cruising’ were recounted as having provided the company of friends, and something interesting to do, but also perhaps a ‘tale’ to be retold as part of the stock of shared experiences of friendship networks:

F1: Remember that time Tara picked us up, it must have been 10 pm, and we snuck out the window when she was just sitting in the car ready to go […] we just wanted the thrill of the chase. [We went] speeding off, we just drove about, but we were just chatting…

F2: It was just so free and easy. It doesn't have to be a destination, it doesn't have to be an A to B.

F1: We just went out cruising about.

F2: We would always go cruising. (A)

On occasion, part of the attraction of a spin was the possibility of it providing not just an outing to remember, but a specifically ‘risky’ one; an escapade with thrills, or an exciting escape from the relative boredom of everyday life:

M: We went on a spin down there before, we were flying about all the country roads doing about 100 mph and then we were doing 100 over Ballybracken Road, flew over a big hill, as soon as we flew over that hill a Jeep just drove straight in front of us and we just missed him. Scary as f**k. Was still good craic but -.

I: And did you get back in the car with that driver?

M: Oh, aye, surely [laughter] I wasn’t gonna walk home! (C)

Perhaps not surprisingly, parents were less keen on the use of cars for simply cruising and socialising in, given their concerns about these kinds of risk taking in particular, and the safety of novice drivers in general:

F1: And then they sort of decided they had they would all travel together in the car, which we weren’t very keen about. But it did, it went on.

F2: You're right, I wouldn't like that I think either. I think I would prefer them on the bus (J)

To mitigate the (well known) risks of both inexperience and deliberate risky behaviour, parents both reported, and were reported as, on occasion placing restrictions on novice drivers:

F1: [My boyfriend’s] parents wouldn't let him be in a car with a younger driver. You know, they would offer to give him a lift or something instead, they wouldn't want him with an inexperienced driver.

M1: My parents were like that as well, they wouldn't want me in the car with someone who had just passed their test. (A)

F1: A friend of mine put a moratorium on her kids travelling together, and I could see why, because she was saying, you know, they could wipe each other out in one fair go. (K)

### Risky driving and driving outside the system

The common use of private cars for entertainment as well as transport, and the tacit social obligations to both provide and accept lifts (particularly in rural areas), led to a number of situations which increased the chance of road danger. In the context of ‘spins’ and entertainment, a few groups (as above) recounted stories of deliberate risk-taking, including fast driving, crossing the national border to avoid police chases, and overcrowded cars. For most groups, though, stories of speeding, joy riding and racing were used to describe ‘other people’s’ behaviour: the dangers of the road were largely seen as generated by risky others, rather than their own inexperience or behaviour. Although admitting to deliberately risk-taking was rare, participants in most groups could recall experiences where they had been in cars that were driven dangerously, suggesting that such incidents were not uncommon:

M: But you take my friend Cathal for example, the man drives like a maniac. The first time I was a passenger in his car we were driving from, I think it was from the pictures and we went into the pub next door for a drink. So he decides to drive well over the speed limit and I end up basically just down the front […]. Whenever he dropped me off I was dribbling and shaking like an idiot, to be honest. (E)

In general, participants were aware of the higher risks for novice drivers: for a few this had deterred them from learning to drive: “it just seems very dangerous” (G). This knowledge was derived from their own experience of other young adults’ driving, and also public information films from the ‘DOE’ (Department of Environment). Such films (broadcast as television adverts built around violent imagery and emotional narratives) were mentioned spontaneously, and reported as effective, in that they were both clearly remembered and taken seriously:

F: I think young people have been really profoundly affected by, like, the DOE ads and stuff that they would show.

F: And they are very graphic, car crashes and things like -

M: - I think they do work, they do work, I think.

F: Oh they do.

M: It puts a lot of people off drink-driving. (A)

M: Definitely takes effect, ‘cos if you were speeding you sorta look at the consequences of what could happen like. You would kill a load of people … It puts you off doing it altogether when you see the adverts. It makes you think. (B)

If most young adult drivers considered that their own driving was safe, they could also describe peers whose driving abilities were not. Here, one participant loyally describes a friend as a ‘really good’ driver, despite also expressing her limited confidence in his ability to keep himself safe on the road:

F: [He] speeds up the road and stuff. It's kind of scary though, because I know he is a really, really, really safe driver and everything but all the crashes happen to young boys. All the crashes here happen to young boys who are speeding and messing about, and it really scares me when he drives really fast. Even though he is really good, he's really good, really responsible, but it's sometimes a bit scary, in case he's going to not come home someday. (G)

This suggests an important point: the social awkwardness of doubting the skills of peers. These stories in general underline the social norm that dangerous driving may be very difficult to challenge within a peer group. Indeed, when participants described situations in which they had accepted lifts even when aware of risks (such as when the car was overcrowded), they generally framed their decision at the time as a necessity: not something they could have chosen not to do. This could be for pragmatic reasons, such as the driver being the only one of a group not to have drunk alcohol, or because there were no other transport options, but underlying these rationales were a sense of the high social costs of declining an offer in a setting in which cars are widely shared resources:

M: I've been in a car with seven people once. […] Everyone needed a lift and it was raining so I just jumped into the back. (E)

Apart from deliberate risk-taking for entertainment, and the situations in which risks were incurred because there was no other apparent choice, there was, more generally, considerable evidence of widespread ‘driving outside the system’, or driving when it would be illegal. One particularly common experience in rural areas was learning to drive on quad bikes or tractors. Legally, tractors can be driven off-road from aged 13, with appropriate training and certification, and on-road from age 16. However, many recalled earlier driving experiences, explaining that some ‘on road’ driving was often essential to manage farm work and useful for picking up the basic skills of driving and road awareness:

M: Well, I probably driving tractors when I was 12 or 13 […] we got experience that way.

F: Behind the wheel in a car or Landrover, I'd probably be 8 or 9 years old. On the farm. On a quad, it would probably be 6 or 7 … Then a JCB [digger], I was a bit older for that, because it was a bit bigger so I was a bit more intimidated by that. So it would be 11 or 12.

M: Your parents probably would have given you a wee crash course. …

F: Yeah. Because the only time I really do drive on the road is when dad's away and the animals need [to be] fed or something. So you know what I mean, there's not too many options. I can't walk it with meal on my back. So it's always necessary, and that's what I'll tell the police when I get stopped. (D)

The ages reported here may be uncommon (and perhaps exaggerated) but this kind of experience of learning to drive on agricultural vehicles in rural areas was typical. There was some uncertainty expressed in discussions about the precise current legal restrictions around, for instance, how far vehicles registered as agricultural vehicles could be used for transport, and at what age. Activities such as “taking the tractor to the shop” (B) were considered not only acceptable but sometimes essential. In contrast, driving while drunk was universally held as unacceptable, with “those who’d taken a drink” identified by participants in all groups as those you wouldn’t accept a lift from:

M: if somebody was really drunk like, I wouldn’t get in. (B)

M: Drink driving has really become, you know, something that nobody does. (J)

However, it was apparent that this normative distaste did not always translate into behaviour: the pragmatic and social incentives for accepting lifts from dangerous drivers also applied to those who had taken a drink. Although driving while drunk was largely described as no longer done, many participants felt that some ‘others’ did still did this. In young people’s groups, these ‘others’ were older drivers, whereas parents identified younger men as the key culprits:

M: It wouldn't really be an issue, no. I mean, I think this generation is actually really careful. If you take the generation from about two generations ago, they're a lot more laissez faire. I know people who would think nothing of having six pints and then getting behind the wheel. But they're good drivers so… and they've never crashed, yet. (E)

F1: Ah it's more young boys, yes

F2: They don’t seem to have the same fear of the law […] we were brought up, you know, in the Troubles^5^ when there was [police] check points anyway. (J)

In practice, then, despite the widespread view that such behaviour was not socially sanctioned, there were suggestions that both driving and accepting lifts might be difficult to avoid even if you knew the driver had had alcohol to drink. It was done for the same reasons as other ‘risky’ driving: that distances were short, roads well known, and there were few alternatives:

M: I know a couple of people that go to the pub and have a couple of pints, or maybe three and go home, drive home […] because they know that it's one straight road home, or it's only around the corner, you know, it's a quiet road (D)

Similar rationales were offered for occasional driving without insurance or a licence for short distances.

### Enforcement and telematics

Relatively high levels of driving ‘outside the system’ (some normatively accepted as inevitable, some disapproved of, but still perhaps common), in the context of the importance of sociability for young adults, has some implications for enforcement of restrictions on novice drivers. In a setting where car travel is necessary, and some high risk driving normalised, there are a number of potential GDL components which might present particular challenges. When views on the proposed scheme were elicited towards the end of the discussions, some suggested that restrictions on taking passengers would be unworkable:

M1: I don’t think it would work

M2: Nor do I

I: Why is that then?

M2: Everybody will still take their mates around as soon as they passed their test (C)

Telematics (in-car devices to monitor driving) are one way in which the enforcement of some driving restrictions (such as curfews and speed limits) can be delegated through technologies. Many young adult drivers and parents had heard of, or had experience of, these technologies, referring to them as ‘the black box’ or ‘the app’. They were typically considered a positive way of reducing insurance premiums and also (by most) as a ‘fair’ way to reward good driving behaviour:

M: It sounds like a good idea. The way they were sort of described on the news one night, which sort of stuck, was people who drive well, this will show they drive well and that will bring their insurance down. But at the same time, if you're a bad driver then you pay the price for being a bad driver. And it's up to you. (G)

F: I had it on mine for the first three months of the insurance last year to lower the insurance. I had to keep a cap on how much I was driving each week for the three months. (E)

Given their concerns about novice drivers, parents described such devices as a reassuring way of delegating their authority:

M: Definitely, if they had them boxes in the car you'd be a lot happier sitting at home.

F: Yeah, you're right. (J)

However, others were ambivalent, or (particularly where they had no experience of the technology), negative. Ambivalence related largely to concerns about ‘fairness’. First, a few thought it was inherently unfair to penalise those who were simply unable, rather than unwilling, to drive in a skilful way. Others pointed to the difficulties of having individual behavioural rewards in a context where cars are to some extent communal resources. Where several people within a household drove a car, and where there may be little real control over who had to be taken as a passenger, concerns were raised about the possibility of linking monitoring to the person:

F: Yes and no, because if someone else jumps into your car who's not on L plates or whatever […] the black box is always on (D)

M: For example, if something's wrong with the car the mechanic will test drive it, and if they speed a bit, that messes up your [recordings]. I thought about the idea of the black box and the app and all but [ …] three of my siblings drive, and I know rightly if they need that car they'll be going out, without insurance, and that car will be away. (G)

More significantly, particularly where such technologies were understood as potentially compulsory, rather than something chosen to bring down insurance premiums, there were also concerns about excess surveillance:

M: Oh no. I would get it ripped out of my car all right. I only started using the phone two or three months ago, because the other tracking device, I don't like it. I just don't - they know where you are at all times. Do you know what I mean? (B)

F1: It's a bit 1984. (Laughter)

F2: It does sound weird. (A)

## Discussion

In summary, in Northern Ireland, cars (and other private vehicles) were viewed as not only essential for accessing work and study, but also for carrying goods as well as people and for accessing commercial, welfare and recreational outlets. Informal car-pooling arrangements were widely reported to facilitate this. A private car could be viewed as a ‘safety zone’ in more urban settings, especially when moving through unfamiliar or unwelcoming areas, and as a comfort zone more generally. Private transport thus has a key role in social life. As has been reported in other studies in UK settings [39], cars also function as an important site of entertainment. The necessity for car travel, the social norms of reciprocity and the importance of cars as a site for socialising led to various accepted situations in which one might ‘drive outside the system’, including some practices that are likely to increase risks of road injury.

Mortality and morbidity data point to the dangers that young drivers pose to themselves and to others, and our qualitative data highlight the potential public health benefits of a GDL programme which reduces exposure to risks for novice drivers such as driving beyond the speed limit, or overcrowding cars. Our findings also point to some potential likely problems in its enforcement. Some participants thought that implementation of elements of the GDL regime might be unenforceable, but problems in enforcing regulations are not a reason for foregoing regulation. In other jurisdictions, non-compliance has been less an issue than predicted [40, 41] although there have been some reports of ignoring restrictions [28].

Importantly, the introduction of a GDL system might reinforce the illegality of what are currently considered (by some) as acceptable driving practices, and help to underline the dangers in using the private car as a ‘recreational tool’. In this way, one important pathway through which a GDL scheme might have the potential to reduce risk is by acting on those psychological factors which are not directly affected by the restrictions imposed or reduced exposure from the scheme itself. Scott-Parker [42], for instance, notes that changing the anticipated psycho-social rewards or punishments associated with risky driving behaviour is challenging, particularly when police enforcement of regulations is likely to be ineffective, and that other potential strategies (such as campaigns using young role models) would be useful in tandem with regulatory change. Our findings suggest that, in this context at least, such campaigns might be effective, given the reported impact of previous road safety campaigns, but also because GDL schemes might change the context in which anticipated rewards and punishments are understood. Given the social difficulties our data suggest in refusing lifts, or not offering them, the restrictions of a GDL scheme may have a major contribution to road safety in providing a socially acceptable way to refuse situations which would otherwise be difficult to avoid, such as overcrowding cars, or accepting lifts with those who drive dangerously. In general, GDL might help to underline the ‘apprentice’ status of young drivers – that is, as people who are not yet fully qualified or fully skilled to drive. This signals to young drivers, their passengers and their parents that passing a practical test does not, in itself, confer driving expertise.

Parents are a vital stakeholder in GDL schemes [43, 44]. Our data suggest that in this setting, parents’ normative expectations are that their children will not only learn to drive, but in doing so will become active members of informal car-pooling networks. Parents are typically responsible for paying for driving lessons, cars and insurance for teenage drivers, and of course also act as role models for driving behaviour [44]. The possibility of a GDL scheme seemingly has considerable traction among parents, some of whom already enforce a rough and ready system by policing their children’s car use. The use of telematics to monitor driving behaviour was welcomed not only by parents, but by many of those young people who had used in-car devices to reduce insurance premiums, although (in the context of a legacy of concern around surveillance) there were some negative associations of monitoring. Telematics are therefore likely to be more acceptable if presented as a way for insurance companies to reward good driving rather than an essential component of any GDL scheme. More generally, GDL schemes may be more readily supported if presented as initiatives to improve or increase the safety of young adults rather than as something that monitors or restricts them.

More specifically, the introduction of insurance-based in-car telematics, which act to potentially reward safe driving (through lower premiums) and sanction risky driving (by both monitoring this, and potentially making insurance prohibitively expensive), have considerable potential for radically changing the rewards and punishments associated with risky driving, by monetising them through insurance premiums.

Our findings have implications for other potential impacts on social wellbeing, beyond injury risk, that need to be considered in any evaluation. Participants commented variously on the use of public transport as a realistic alternative to ‘the car’. As well as being regarded as of poor quality, and inconvenient (especially in bad weather and late at night), use of public transport, and walking or cycling, was seen as socially inappropriate. Such transport could also be viewed as unsafe, leaving users open to possible threats of violence and intimidation. Private cars remain important in the everyday life of young people in areas of thinly dispersed settlements [45], beyond providing a functional mode of transport from A to B. In Northern Ireland, there was a widespread normative expectation that transport requirements were a communal responsibility, primarily within households, but also more widely across peer groups. ‘The car’ therefore underpins social networks and acts as an important cog in systems of social capital. Restrictions on how far newly licenced drivers can contribute to this network (if, for instance, they restrict passengers) may have a far greater impact on rural novice drivers than those in area with better public transport provision. Over and above that, there remains in this setting a symbolic edge to ‘the car’ as a marker of independence and perhaps of social status. The corollary of this is that relatively safe public transport is unlikely (yet) to be considered an attractive alternative for young people. Alternatives such as cycling were unappealing; and (like taking the bus) were perceived to be risky. Reductions in reliance on private car transport and its association with ‘adult independence’ can be achieved with shifts in availability and perceptions of public transport for young adults, as the example of London has shown [46], but this does require considerable investment in infrastructure, and may be unrealistic in thinly populated areas.

Our data suggest that there is already considerable driving outside the system where restrictions on driving were seen as unrealistic. As in other rural areas [45], some of this is widely tolerated as part of the necessary compromises that need to be made in order to manage everyday life, such as allowing children to drive tractors short distances. If GDL schemes were overly restrictive, there may be incentives to exacerbate the use of agricultural vehicles for these purposes. However, these data also show that norms about what is reasonable are clearly malleable, given the almost universal reporting of changed views on drink driving, and the widespread perceptions that public information films on the dangers of driving were both credible, and effective. This suggests that good quality publicity on proposed GDL schemes would be essential to reinforce the need for restrictions, and help change social views on the role of cars as sources of entertainment.

These findings have implications for how GDL interventions will potentially impact on the public health, and should therefore be evaluated. Whilst injury rates are the major public health impact (with consequences for young people into the future), it is important that any evaluation of GDL schemes does not simply look at road injury rates for licensed drivers. These are unacceptably high for young drivers in Northern Ireland, and any interventions to reduce them are to be welcomed, but there are a number of other likely implications for the health and wellbeing of young drivers and their passengers which need to be taken into account to assess whether schemes are beneficial overall for public health, and to ensure that the knowledge gained can be used by policy makers in other jurisdictions.

A first implication is that injury rates need to be evaluated in terms of both the reductions overall, and the reductions in number of licensed drivers, to assess whether the impact is in part through reducing or delaying licensing in young adults; or conversely (in a setting where there is much car-pooling) there are increases in the number of drivers or cars, if car-pooling between young drivers becomes less available as a result of GDL constraints. Injury rates for all transport modes also have to be considered, to ensure that restrictions do not simply shift travel to other, potentially more risky, modes such as motorbikes or cycling. Given the reliance on private car use in rural areas, and the extensive car-sharing to which young adults were expected to contribute as soon as they were able, there is evidence that being able to provide lifts for others is a crucial element in the social economy of rural life, and the broader potential impact on social exclusion has to be considered to assess the impact of GDL on equity.

The strengths of this study were: the use of natural groups to access detailed accounts of practices, including those involving risk-taking and driving outside the system, and the timing, which enabled us to identify social norms around driving at a point before there was much public awareness of the proposed GDL scheme. The limitations of our small sample size (designed to elucidate key pathways, not identify the prevalence of health impacts of driving for young adults) are that this did not allow a full exploration of how the pathways we identify are likely to vary across contexts of rurality, deprivation, and so on. In particular, we included few parents, and more data would be needed to understand the full range of approaches to enforcing restrictions, training in driving and modelling driving skills. Once GDL is introduced, if parents’ roles as supervising drivers extend to facilitate longer training periods, there may be unanticipated benefits from teenagers sharing time with parents, for instance [47]. Although the key pathways between car use and public health impact identified here are likely to be generalizable to other settings in which cars are shared in households, some factors identified in our results (such as concerns over surveillance) may be particular to this setting. The role of in-car telematics in changing driving behaviour in this age group is potentially a major one, and likely to have an impact independently of any GDL schemes. Any future evaluation of GDL will, therefore, have to include an assessment of the role of insurance company policies and technologies on risks of road injury. As telematics technologies are rolled out, they may also facilitate novel approaches for evaluating other interventions.

## Conclusion

GDL schemes have the potential to reduce road injury in novice drivers through reducing their exposure to high risk situations and increasing their skills through longer mandated periods of training. A proposed scheme in Northern Ireland, but not other countries in the UK, provides an opportunity to undertake an evaluation of the public health impact of GDL in a European context that utilises a comparative natural experiment design. Our data suggest evaluation of this scheme should take account of injury impacts on all classes of road user, and also any (even short term) implications of passenger and night-driving restrictions for transport exclusion of young people, particularly in rural areas where a shared economy of lift-provision is essential for access to work and social life. We also suggest evaluating the impact on social norms around car sharing and risky driving. If car sharing becomes less acceptable, there are potential public health losses from increased numbers of sole-occupied cars. Future evaluations will need to take into account the impact of increased adoption of in-car telematics on reducing road injury in young adults.

## Abbreviations

DfT: Department for Transport
DOE: Department of Environment
GDL: Graduated driver licensing
NIMDM: Northern Ireland multiple deprivation measure
UK: United Kingdom

## Footnotes

1 Comparable figures for car ownership are difficult to access, but in general European countries have lower rates: see for instance data comparing crude numbers of vehicles per person in population: http://www.nationmaster.com/country-info/stats/Transport/Motor-vehicles. Accessed on 6 June 2017.

2 Estimated from data available from https://www.doeni.gov.uk/publications/dva-enforcement-statistics-2015-2016-quarter-2 and http://www.nisra.gov.uk/demography/default.asp20.htm. Accessed on 6 June 2017.

3 Estimated from data from Department for Infrastructure (2016) Northern Ireland Transport Statistics: vehicle registrations 2014–2015 Available from https://www.infrastructure-ni.gov.uk/publications/northern-ireland-transport-statistics-2014-2015. Accessed on 6 June 2017.

4 The 12th July public holiday commemorating a Protestant victory can be a period of heightened sectarian tensions.

5 The ‘Troubles’ is used as a local colloquialism to describe over thirty years of violent conflict in Northern Ireland which came to an end with the signing of the Good Friday Agreement in 1998.
